# Titanium Dioxide Nanoparticle Circulation in an Aquatic Ecosystem

**DOI:** 10.1007/s11270-018-3852-8

**Published:** 2018-06-12

**Authors:** Monika Asztemborska, Małgorzata Jakubiak, Romuald Stęborowski, Ewelina Chajduk, Grażyna Bystrzejewska-Piotrowska

**Affiliations:** 1 Isotope Laboratory, Faculty of Biology, University of Warsaw, Miecznikowa 1, 02-096 Warsaw, Poland; 2 Department of Analytical Chemistry, Institute of Nuclear Chemistry and Technology, Dorodna 16, 03-195 Warsaw, Poland

**Keywords:** Nanoparticles, TiO_2_, *Elodea canadensis*, *Danio rerio*, Aquatic ecosystem

## Abstract

Nanotechnology is a dynamically developing field of scientific and industrial interest across the entire world, and the commercialization of nanoparticles (NPs) is rapidly expanding. Incorporation of nanotechnologies into a range of manufactured goods results in increasing concern regarding the subsequent release of engineered NPs into the environment. One of the biggest threats of using NPs is the transfer and magnification of these particles in the trophic chain. The aim of the studies was the evaluation of the distribution of TiO_2_ NP contamination in the aquatic ecosystem under laboratory conditions. Bioaccumulation of TiO_2_ NPs by plants (*Elodea canadensis*) and fish (*Danio rerio*) in the source of contamination was investigated. The studies were focused on the consequences of short-term water contamination with TiO_2_ NPs and the secondary contamination of the components of the investigated model ecosystem (plants, sediments). It was found that in the fish and the plants exposed to NP contamination, the amount of Ti was higher than in the control, indicating an effective bioaccumulation of NPs or ions originating from NPs. It was clearly shown that the NPs present in the sediments are available to plants and fish. Additionally, the aquatic plants, an important trophic level in the food chain, can accumulate NPs and be a source of NPs for higher organisms. It was concluded that even an incidental contamination of water by NPs may result in long-term consequences induced by the release of NPs.

## Introduction

Nanotechnology, defined as the understanding and control of matter at dimensions of roughly 1–100 nm, is a dynamically developing field of scientific and industrial interest in the entire world. Because of its exceptional potential, there has been a drastic increase in investment in nanotechnology research and development worldwide. Nanoparticles (NPs) are increasingly used in many types of consumer products. Among the most commonly used are titanium oxide NPs (titania NPs, TiO_2_ NPs); for example, they are present in paints, cements, sunscreen, car cosmetics, catalysts, ultraviolet protection devices, and batteries (Biswas and Wu 2005; Mann 2006).

In 2005, nanoscale TiO_2_ global production was 2000 tons (US EPA 2009). By 2010, the nanosize TiO_2_ production grew by 250% and reached 5000 t, and it is expected to continue to increase until at least 2025 (Landsiedelet et al. 2010). After use, NP-based products can enter the sewage system and subsequently enter the environment as treated effluents discharged to the surface waters or biosolids applied to the agricultural land, incinerated wastes, or landfill solids (Weir et al. 2012). Therefore, it is assumed that these NPs are getting into the aquatic environment (Guzman et al. 2006; Nowack and Bucheli 2007; Kaegi et al. 2008), and they can be bioaccumulated through the food chain.

Some work has been done on the toxicity of NPs to aquatic organisms. Hund–Rinke and Simon (2006) examined the ecotoxic effect of TiO_2_ NPs on algae and daphnids. The effective concentration (EC50s) of TiO_2_ NPs (25 nm) in *Desmodesmus subspicatus* is 44 mg/L. Ma et al. (2012) estimated LC_50_s greater than 500 mg TiO_2_/L and 155 mg TiO_2_/L for *Daphnia magna a*nd *Japanese medaka*, respectively. Heinlaan et al. (2008) have shown that 20 g TiO_2_/L induced 60% mortality of *D. magna*. The concentration of 20 g TiO_2_/L was not acutely toxic for bacteria *Vibrio fischeri* and crustaceans *Thamnocephalus platyurus*. Chen et al. (2011) showed the adverse effect of TiO_2_ NPs at concentrations of 1.0–7.0 mg/L on *Danio rerio*, resulting in the inhibition of growth and a decrease in the liver weight ratio of *D. rerio*. The gills displayed histopathological changes including the thickening of edema and the gill lamellae. TiO_2_ NPs could translocate among organs and pass through the blood–brain and the blood–heart barrier after long-term exposure. Rocco et al. (2015) proved that TiO_2_ NPs induce *D. rerio* DNA damage when the concentration was 10 μg/L for 14 and 21 days of treatment. Chronic exposure of *D. rerio* to 0.1 mg/L of TiO_2_ NPs weakened its reproduction; there was a 29.5% reduction in the number of *D. rerio* eggs after 13 weeks of exposure (Wang et al. 2011). Xiong et al. (2011) have found that the size distribution of NPs was similar to that of the bulk particles in suspension, and the acute toxicity of 124.5 mg/L of TiO_2_ NPs (96-h LC_50_) to *D. rerio* was greater than that of the bulk TiO_2_, which was essentially non-toxic. The bioavailability and toxicity of TiO_2_ NPs can be affected by environmental factors, including presence of humic acids (Yang et al. 2013; Bystrzejewska–Piotrowska et al. 2012).

One of the biggest threats of using NPs is the transfer and magnification of these particles in the trophic chain. The studies of silver bioaccumulation by freshwater larvae of the insect *Chironomus* (diptera: *Chironomidae*) and *D. rerio* fish have shown that the amount of silver uptake by fish depends on the contamination source (Asztemborska et al. 2014). Larvae that have accumulated silver NPs can be a source of NPs in fish and certainly higher organisms. Only a few studies have been conducted in this area for TiO_2_ NPs. Zhu et al. (2010) established a simple model of a freshwater food chain including low (*D. magna*) and high (*D. rerio*) trophic-level organisms. The results proved that TiO_2_ NPs can transfer from *D. magna* to *D. rerio* by dietary exposure, but the biomagnification of NPs was not observed; the values of the biomagnification factors (BMFs) in this study (0.024 and 0.009) were all less than 1 (Zhu et al. 2010b). Additionally, the toxicity of TiO_2_ NPs was examined, showing minimal toxicity to *D. magna* within the traditional 48-h exposure time; however, high toxicity was observed when the exposure time was extended to 72 h (Zhu et al. 2010a).

Considering a variety of factors affecting the bioavailability and the toxicity of NPs, the evaluation of the environmental hazards and risks associated with release of NPs to the natural environment must be based on the results of ecotoxicological studies. Our knowledge of nano*-*TiO_2_ bioaccumulation in aquatic organisms in complex ecosystems is limited and must be extended.

The aim of the present study was to evaluate the distribution of TiO_2_ NP contamination in the model aquatic ecosystem. For this purpose, bioaccumulation of TiO_2_ NPs by plants (*Elodea canadensis*) and fish (*Danio rerio*) depending on the source of contamination was investigated. Different analytical techniques were performed to determine the amount of titanium in plants and fish tissues and to indentify its distribution in fish bodies. The consequences of short-term and long-term water contamination with TiO_2_ NPs for aquatic ecosystem were discussed.

## Materials and Methods

### Materials

**Fig. 1:**
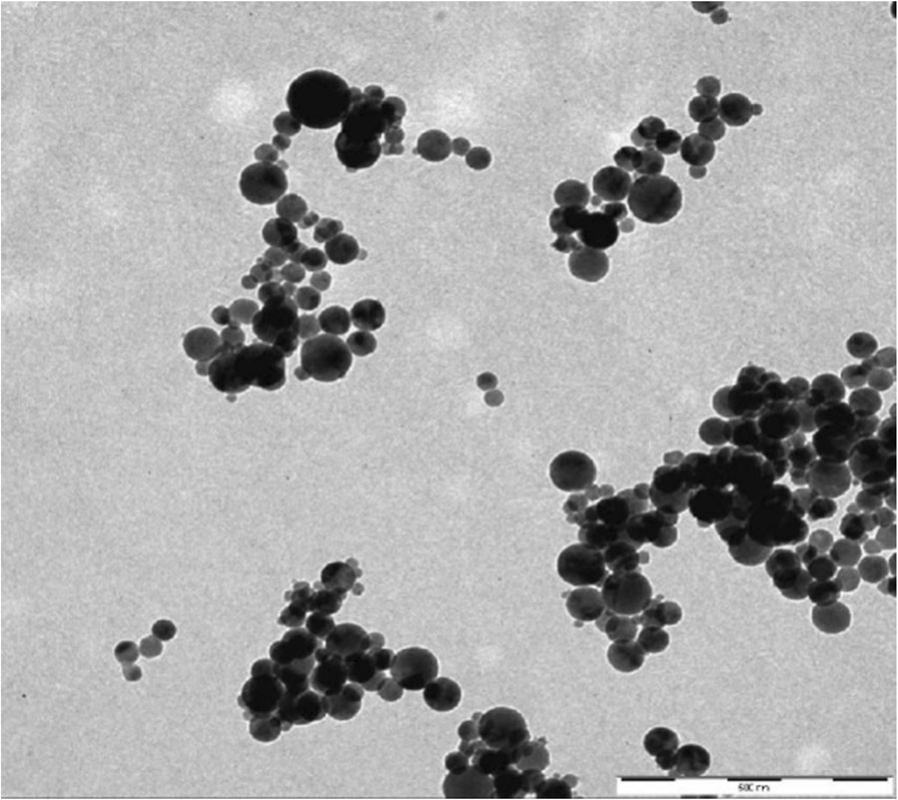
Transmission electron micrographs of TiO_2_ nanoparticles (suspended in water) used in the experiment

TiO_2_ NPs (nanopowder, < 100 nm) were purchased from Sigma-Aldrich. The particle size below 100 nm and spherical morphology of nanoparticles were characterized in 1-mmol/L water suspensions using a TEM LEO 912AB transmission electron microscope (Zeiss) equipped with a Proscan high-speed slow-scan CCD camera (Fig. 1).

Adult male *D. rerio* fish (line species ABTL) were obtained from the Zebrafish Core Facility (ZCF) at the International Institute of Molecular and Cell Biology in Warsaw, Poland. *Elodea canadensis* plants used in the experiment came from the Botanical Garden of the Warsaw University, Poland.

### Exposure of *E. canadensis* and *D. rerio* to TiO_2_ NPs

**Fig. 2:**
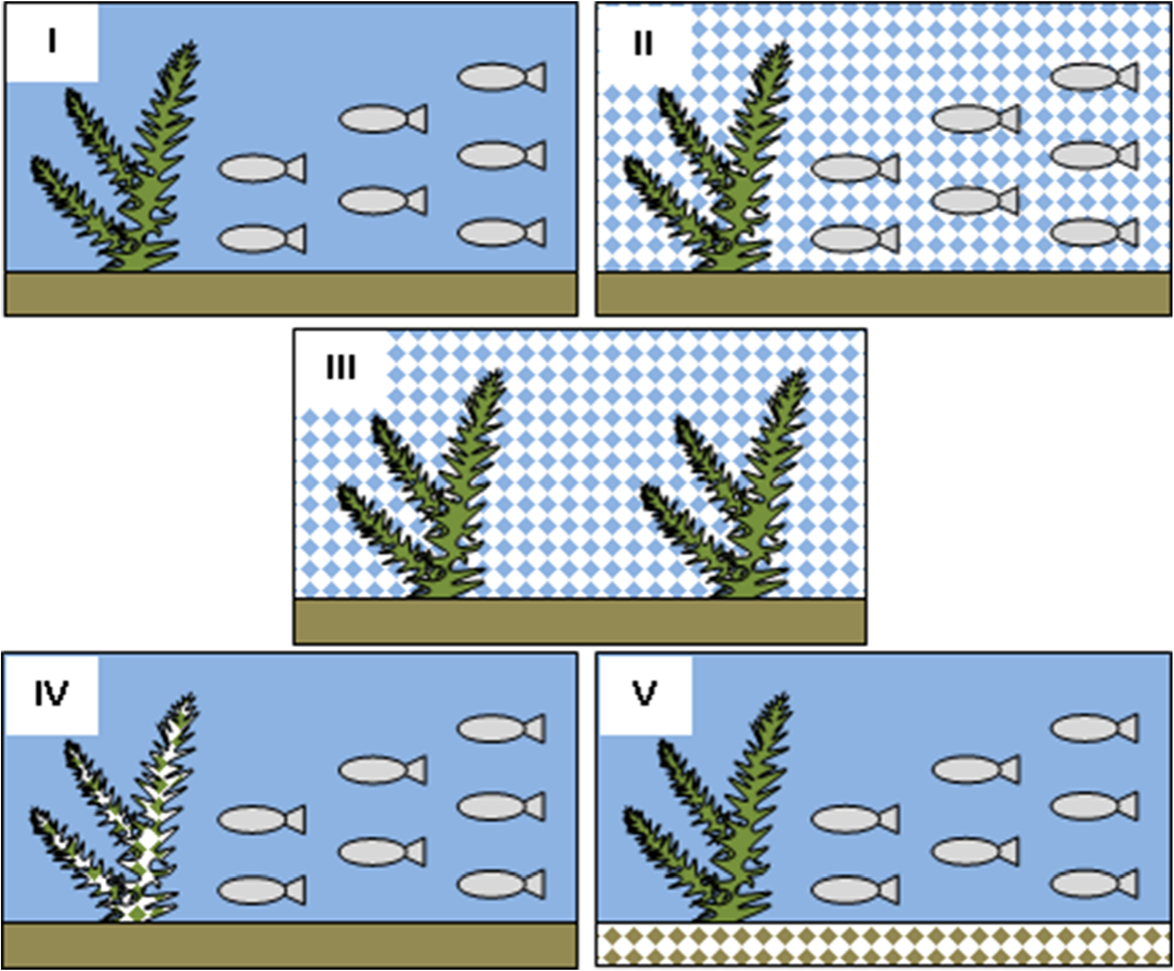
Experiment diagram. Texture indicates the objects that were contaminated with TiO_2_ NPs. Research variant: I—control; II—10 mg/L TiO_2_ in water; III—10 mg/L TiO_2_ in water, without fish; IV—TiO_2_ in plants from variant III; and V—TiO_2_ in the aquarium base from variant III

Exposure of *E. canadensis* and *D. rerio* to TiO_2_ NPs was performed in 10-L aquariums. The water temperature was 24 °C, and the organisms were subjected to a photoperiod of 14 h of light and 10 h of darkness. *Danio rerio* (seven fish per aquarium) and *E. canadensis* (15 g of fresh fish plant per aquarium) were exposed for 2 weeks to TiO_2_ NPs from different sources. The experimental variants included the following: (I) control, (II) plants and fish placed in an aquarium with TiO_2_ NPs at a concentration of 10 mg/L, (III) doubled portion of plants placed in an aquarium with TiO_2_ NPs at a concentration of 10 mg/L, (IV) a portion of plants from the third experimental variant placed together with a fish in the aquarium (contaminated plants were a source of NPs), and (V) a base from the third experimental variant placed together with plants and fish in the aquarium (contaminated base was a source of NPs). Details of the research variants are presented in Fig. 2. Each variant of the experiment was performed in three replicates.

After the exposure, the fish and the plants were separated from the water and rinsed with distilled water. Further, tricaine (3-aminobenzoic acid ethyl ester) from Sigma was used for anesthetizing the fish. Next, the samples were frozen at − 24 °C and freeze-dried and homogenized before further analysis.

### Determination of Total Ti Content

For the determination of the Ti content in *D. rerio* and *E. canadensis*, about 100 and 250 mg of a dried, homogenized material, respectively, was digested with a mixture of 5 mL HNO_3_, 2 mL H_2_O_2_, 1 mL HF, and 6 mL H_3_BO_3_ by using a microwave laboratory system, Multiwave 3000 (Anton Paar, Germany). After digestion, the samples were quantitatively transferred with 2% HNO_3_ into volumetric flasks (25 mL) and analyzed by inductively coupled plasma mass spectrometry, ICP MS (ELAN 6000 ICP mass spectrometer, PE SCIEX, Concord, Canada).

### Determination of Ti Deposition in the Fish Tissues

The imaging of the NP and metal deposition in the fish tissues was carried out using an X-ray analysis, laser ablation inductively coupled plasma mass spectrometry (LA-ICP MS), and transmission electron microscopy (TEM).

In the X-ray analysis of the metal location in the fish body by the Carestream In Vivo MS FX PRO system, one fish from each research variant was selected. The following settings were used for obtaining the X-ray images: illumination source X-ray 20 kVp, exposure time 30 s, no excitation filter, no emission filter, no binning, f-stop 2.8, focal plane 13.1 mm, and field of view 27.80 mm. Next, the same fish was analyzed for metal deposition by using an ELAN 6000 ICP mass spectrometer (PE SCIEX, Concord, Canada). The titanium content in fish was determined in the selected 5-mm parts of the fish body (the ventral part, operculum, caudal fin, and below the dorsal fin) in the cross section of the ventral part of the fish.

More detailed studies of TiO_2_ NP deposition in fish cells were conducted using TEM. For this purpose, certain small parts of the gills, mussels, and the digestive system were fixed with a 2.5% glutaraldehyde/cacodylic buffer and incubated for 1 h and then washed in 0.1 M cacodylic buffer. Next, the samples were post-fixed in 1% OsO_4_ in ddH_2_O for 12 h and washed three times in ddH_2_O. After post-fixation, the samples were dehydrated through a graded series of EtOH (50%, 10 min; 70%, 24 h; 90%, 10 min; 96%, 10 min; anhydrous EtOH, 10 min; acetone, 10 min) and infiltrated with EPON resin in acetone (1:3, 30 min; 1:1, 30 min; 3:1, 2 h), infused twice for 24 h in pure EPON resin, and polymerized for 48 h at 60 °C. Next, 60-nm sections were prepared with ultramicrotome RMC MT-X (Tucson, USA) and were examined on a Libra 120 electron microscope (Carl Zeiss, Germany). The images were captured by the slow-scan CCD camera using WinTEM™ Graphical Use Interface.

### Statistical Analysis

Results were expressed as mean ± standard deviation, *n* = 3. Analysis of variance (ANOVA) was used to determine statistical significance of the differences between values. Statistical significance for all tests was set at *P* ≤ 0.05.

## Results and Discussion

### Bioaccumulation of Ti in *D. rerio* and *E. canadensis*

The Ti content in *D. rerio* and *E. canadensis* determined using ICP MS is presented in Table 1. In all the samples of the fish and the plants exposed to NP contamination, the amount of Ti was higher than in the control, indicating an effective bioaccumulation of NPs or ions originating from NPs.

**Table 1:** Titanium contents in fish and plants exposed for 2 weeks to TiO_2_ NPs

Research variant	Ti content (g kg^−1^ d.w.)	
	*Danio rerio*	*Elodea canadensis*
I	0.06 ± 0.01	0.03 ± 0.01
II	0.21 ± 0.01	3.50 ± 0.53
III	n/a	1.85 ± 0.20
IV	0.09 ± 0.01	0.72 ± 0.12
V	0.13 ± 0.01	2.02 ± 0.09

Research variant: I—control; II—10 mg/L TiO_2_ in water; III—10 mg/L TiO_2_ in water, without fish; IV—TiO_2_ in plants from variant III; and V—TiO_2_ in the aquarium base from variant III

The highest content of Ti (3.5 g/kg of dry weight (d.w.)) was found in plants cultivated in water contaminated with TiO_2_ NPs (variant I). This can be an effect of the bioaccumulation of NPs or their deposition on the plant surface. In the *E. canadensis* from experimental variant III, the amount of Ti was ca. 50% lower because of the doubled number of plants in the container. After transferring the plants to the non-contaminated water (experimental variant IV), the amount of Ti was reduced of ca. 60%. This suggests that NPs were weakly bounded or adsorbed at the plant surface, and after a transfer to the uncontaminated environment, they moved into the water. A relatively high content of Ti (ca. 2 g/kg d.w.) was determined in the samples of plants cultivated in the aquarium with the base contaminated with TiO_2_ NPs. This clearly shows that the NPs present in the sediments were available to plants. Irrespective of the source of the TiO_2_ NP contamination, *E. canadensis* had elevated content of Ti as compared to the control samples. This indicated that the aquatic plants, an important trophic level in the food chain, can accumulate NPs and be a source of NPs for higher organisms.

These results were in agreement with the results of Ti bioaccumulation in *D. rerio*. The highest content of Ti was determined in fish exposed to TiO_2_ NPs in water, while the lowest concentration was found in organisms staying together with the contaminated plants (variant IV). The increase in the Ti concentration in different organs of *D. rerio* as an effect of water contamination with TiO_2_ NPs was previously documented by Chen et al. (2011). In fish exposed to NPs at a concentration of 7 mg/L, after 6 months of exposure, the Ti content was in the range of 45–60 μg/kg in the brain, liver, and heart, and the highest value was determined in gills, ca. 100 μg/kg. In the present study, the Ti content determined in *D. rerio* was considerably higher; however, the entire fish body was analyzed in the experiments.

Fish can accumulate NPs in their gills and/or body shell or by feeding on contaminated plants. We can also expect that some adsorption or TiO_2_ NPs on the fish body take place. In the variant with contaminated water, the TiO_2_ NP concentration available for organisms was obviously the highest, resulting in the highest bioaccumulation by both organisms. We expect the bioaccumulation by the gills and body shell and the adsorption of TiO_2_ NPs on the fish body to be the dominant processes responsible for the observed effect. In experimental variant IV, the only source of Ti for fish was plants. The Ti content in plants was lower than in the case of water contamination. Here, we concluded that feeding on the contaminated plants was the main process of Ti bioaccumulation by fish. A significant amount of Ti was also accumulated by fish in experimental variant V with the aquarium base contaminated with TiO_2_ NPs. Therefore, it was obvious that in water, TiO_2_ NPs tend to sediment at the bottom of the container. A relatively high content of Ti in fish bodies as compared to that in the case of water contamination (ca. 40% lower) implied that Ti was still available for organisms. These results clearly show that the bioaccumulation of Ti by *D. rerio* depends on the source of the NP contamination, which is in agreement with the results of previous studies on the bioaccumulation of silver NPs by the same fish (Asztemborska et al. 2014). We found that the silver content in *D. rerio* living in water and containing NPs was 110-fold lower than that in organisms fed with contaminated *Chironomid* larvae, but remained in the digestive system (Asztemborska et al. 2014).

### Metal Location in *D. rerio*

#### X-Ray Analysis

**Fig. 3:**
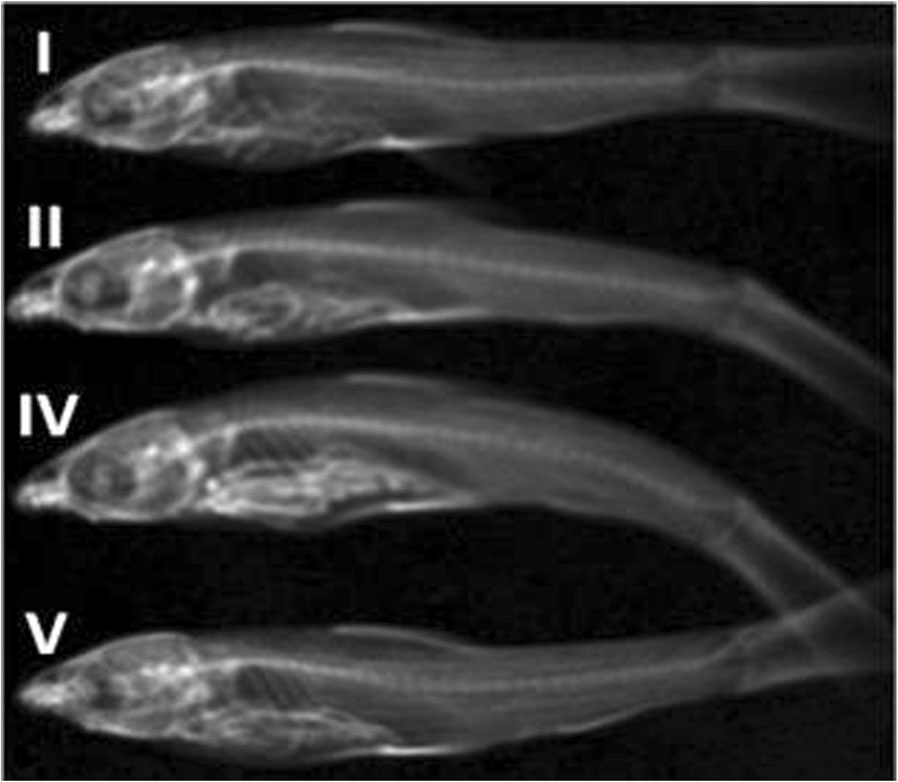
Metal location in fish body obtained by X-ray. Research variant: I—control; II—10 mg/L TiO_2_ in water; III—10 mg/L TiO_2_ in water, without fish; IV—TiO_2_ in plants from variant III; and V—TiO_2_ in the aquarium base from variant III

The next step of the studies was the analysis of the Ti location in the fish bodies. X-ray images showed that the metal was accumulated in the ventral part of the fish body in each research variant (Fig. 3). Irrespective of the lowest total Ti bioaccumulation, the highest concentration was observed in the *D. rerio* originating from experimental variant with contaminated plant as TiO_2_ NP source. This confirms the conclusion that feeding on contaminated plants is one of the main processes resulting in Ti bioaccumulation by fish under the considered conditions. However, a significant concentration was determined in the *D rerio* from the variants with TiO_2_ NPs containing water or from the aquarium base. This indicates that contaminated food is an important source of NPs for fish.

#### LA-ICP MS

**Fig. 4:**
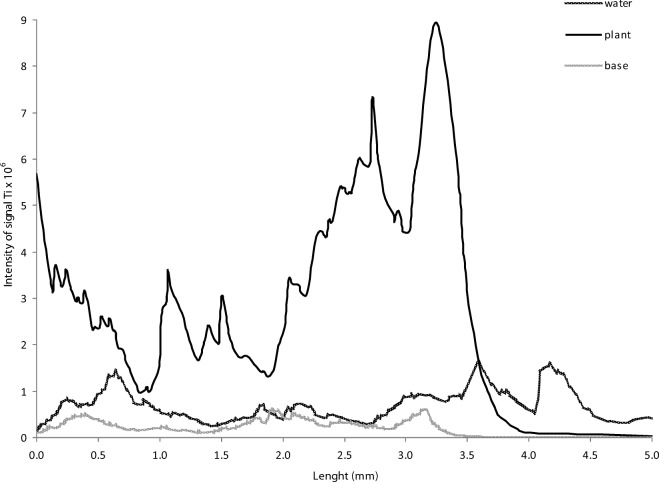
Intensity signal of titanium in the ventral part of the fish (LA-ICP MS)

**Fig. 5:**
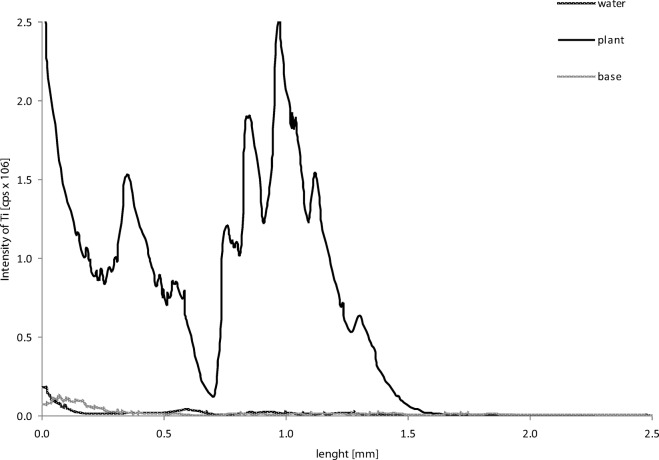
Intensity signal of titanium in deeper ventral part of the fish (LA-ICP MS)

On the basis of the results obtained from the X-ray images, the measuring points for LA-ICP MS were determined. The Ti concentration in the ventral part of the fish from each research variant is presented in Fig. 4. The Ti level in the fish bodies from all the experimental variants was significantly higher than that in the control. The results obtained by the X-ray analysis have been confirmed: the highest Ti concentration was found in *D. rerio* originating from the experimental variant with contaminated plants as the source of the TiO_2_ NPs. To confirm the presence of Ti in the digestive system, an analysis of the cross section of the ventral part of the fish was done (Fig. 5). The results revealed a very high Ti content in the *D. rerio* in the aquarium with the TiO_2_ NP-contaminated plants. In the rest of the examined samples, the Ti content was lower; however, it was still significantly higher than that in the control fish. This confirms that plants contaminated with NPs can be a very efficient source of contamination for higher organisms.

#### TEM

**Fig. 6:**
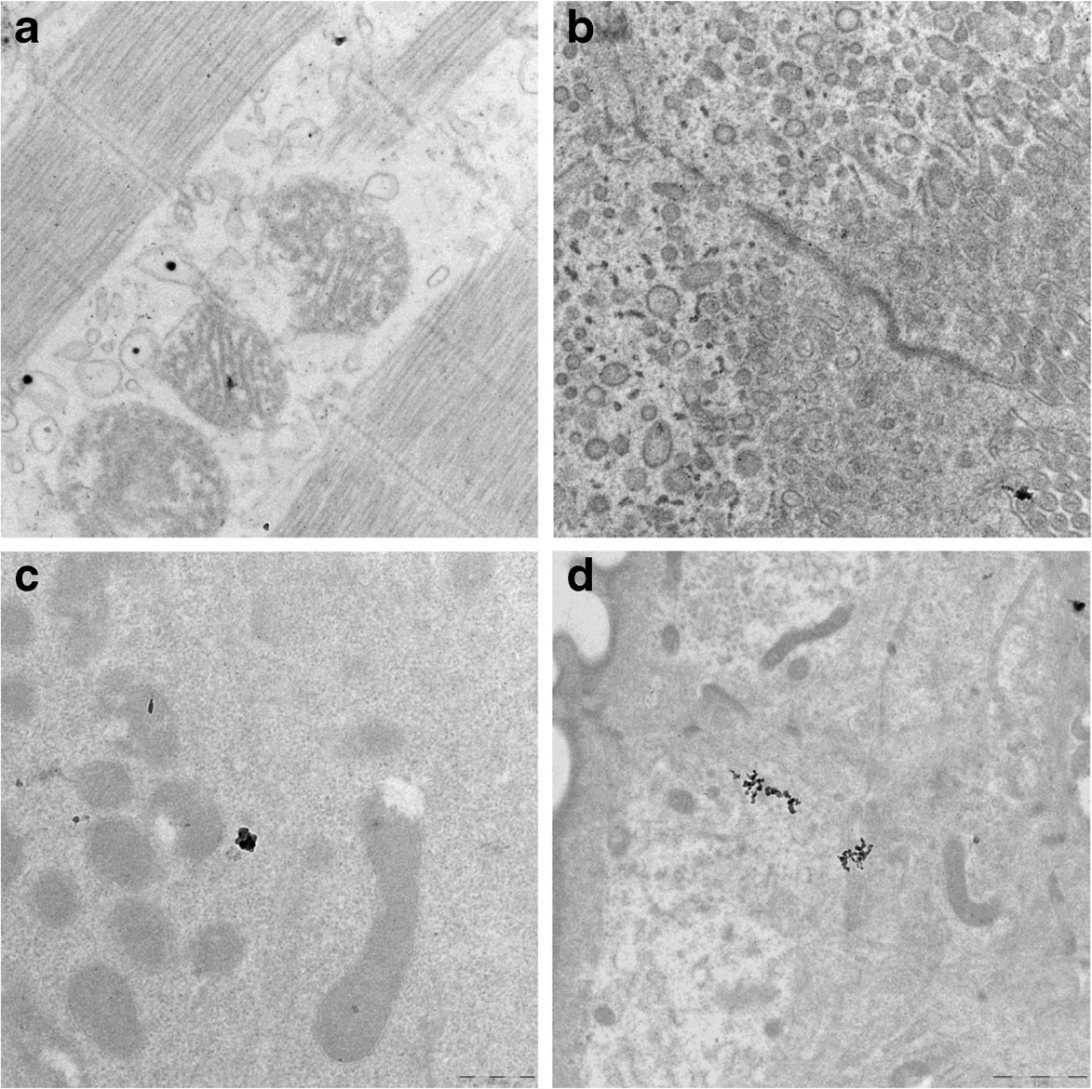
Transmission electron micrographs of TiO_2_ nanoparticles in selected tissues of *Danio rerio*. **a** Mussels, variant II. **b** Intestine, variant II. **c** Intestine, variant III. **d** Gills, variant II

The highest content of Ti in the ventral part of the fish body was determined in *D. rerio* from experimental variant III (contaminated food); however, the highest total bioaccumulation of Ti was found in variant II (water contamination). This is an effect of the high concentration of Ti in a limited part of the digestive system of the *D. rerio* feeding on contaminated plants and a more evenly distributed Ti content in the body of the fish in the rest of the variants. Imaging of the Ti content in the operculum, caudal fin, and below the dorsal fin by LA-ICP MS was then performed. The results were inconclusive but seemed to confirm the above conclusion. The analysis of gills, mussels, and the intestine by TEM revealed TiO_2_ NPs in fish cells (Fig. 6). In the *D. rerio* exposed to NPs in water, some TiO_2_ NPs were found in the mussels and gills (Fig. 6a, d, respectively). TiO_2_ NPs were also found in the intestine of *D. rerio* exposed to NPs in the form of contaminated plants (Fig. 6c); however, some aggregates of TiO_2_ NPs could be seen on micrographs of fish from the variants with contaminated water.

## Conclusion

The aim of this study was to evaluate the distribution of TiO_2_ NP contamination in an aquatic ecosystem. It was found that (i) plants *E. canadensis* and fish *Danio rerio* exposed to TiO_2_ nanoparticle contamination effectively accumulate NPs or ions originating from NPs; (ii) aquatic plants, an important trophic level in the food chain, can be a source of NPs for higher organisms; and (III) NPs stored in the sediment can still interact with organisms and be available for plants and fish.

**Fig. 7:**
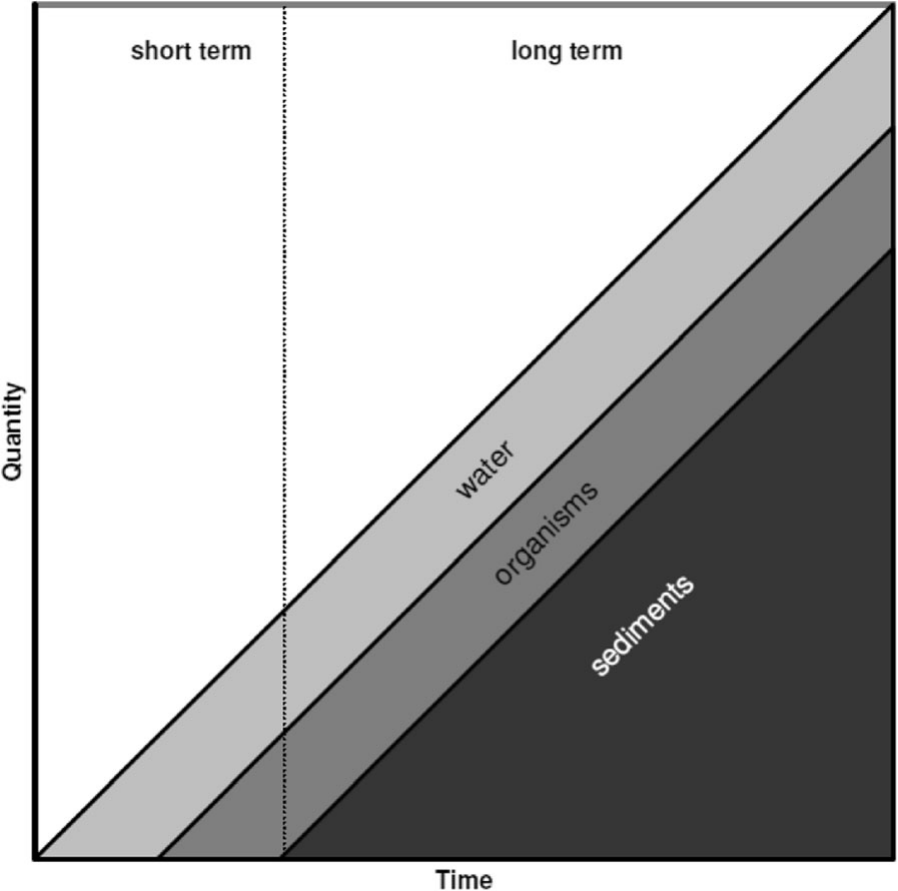
Short- and long-term contamination of an aquatic environment with nanoparticles—an interpretation of Amara’s law

It was concluded that even an incidental contamination of water by NPs may result in long-term consequences induced by the release of NPs. The interpretation of Amara’s law in the view of the growth of nanotechnologies made by Bystrzejewska-Piotrowska et al. (2009) can be applied for the estimation of short- and long-term consequences of water system contamination by NPs (Fig. 7). A further investigation of such parameters as the level and the intensity of release of NPs from the sediments to the water and the organisms living in aquatic ecosystems is required.

## References

[CR1] Asztemborska M, Jakubiak M, Książyk M, Stęborowski R, Polkowska-Motrenko H, Bystrzejewska-Piotrowska G (2014). Silver nanoparticle accumulation by aquatic organisms—neutron activation as a tool for the environmental fate of nanoparticles tracing. Nukleonika.

[CR2] Biswas P, Wu CY (2005). Nanoparticles and the environment. Journal of the Air & Waste Management Association.

[CR3] Bystrzejewska-Piotrowska G, Golimowski J, Urban PŁ (2009). Nanoparticles: Their potential toxicity, waste and environmental management. Waste Management.

[CR4] Bystrzejewska-Piotrowska G, Asztemborska M, Giska I, Mikoszewski A (2012). Influence of earthworms on extractability of metals from soils contaminated with Al2O3, TiO2, Zn, and ZnO nanoparticles and microparticles of Al2O3. Polish Journal of Environmental Studies.

[CR5] Chen J, Dong X, Xin Y, Zhao M (2011). Effects of titanium dioxide nano-particles on growth and some histological parameters of zebrafish (*Danio rerio*) after a long-term exposure. Aquatic Toxicology.

[CR6] Guzman KAD, Finnegan MP, Banfield JF (2006). Influence of surface potential on aggregation and transport of titania nanoparticles. Environmental Science & Technology.

[CR7] Heinlaan M, Ivask A, Blinova I, Dubourguier HC, Kahru A (2008). Toxicity of nanosized and bulk ZnO, CuO and TiO2 to bacteria *Vibrio fischeri* and crustaceans *Daphnia magna* and *Thamnocephalusplatyurus*. Chemosphere.

[CR8] Hund-Rinke K, Simon M (2006). Ecotoxic effect of photocatalytic active nanoparticles (TiO2) on algae and daphnids. Environmental Science and Pollution Research International.

[CR9] Kaegi R, Ulrich A, Sinnet B, Vonbank R, Wichser A, Zuleeg S, Simmler H, Brunner S, Vonmont H, Burkhardt M, Boller M (2008). Synthetic TiO2 nanoparticle emission from exterior facades into the aquatic environment. Environmental Pollution.

[CR10] Landsiedel R, Ma-Hock L, Kroll A, Hahn D, Schnekenburger J, Wiench K, Wohlleben W (2010). Testing metal-oxide nanomaterials for human safety. Advanced Materials.

[CR11] Ma H, Brennan A, Diamond SA (2012). Phototoxicity of TiO_2_ nanoparticles under solar radiation to two aquatic species: *Daphnia magna* and *Japanese medaka*. Environmental Toxicology and Chemistry.

[CR12] Mann, S., 2006. Nanotechnology and Construction Nanoforum Report. Institute of Nanotechnology, Stirling.

[CR13] Nowack B, Bucheli TD (2007). Occurrence, behavior and effects of nanoparticles in the environment. Environmental Pollution.

[CR14] Rocco L, Santonastaso M, Mottola F, Costagliola D, Suero T, Pacifico S, Stingo V (2015). Genotoxicity assessment of TiO_2_ nanoparticles in the teleost *Danio rerio*. Ecotoxicology and Environmental Safety.

[CR15] United States Environmental Protection Agency, 2009. External review draft–nanomaterial case studies: nanoscale titanium dioxide in water treatment and in topical sunscreen. EPA/600/R-09/057.

[CR16] Wang J, Zhu X, Zhang X, Zhao Z, Liu H, George R, Wilson-Rawls J, Chang Y, Chen Y (2011). Disruption of zebrafish (*Danio rerio*) reproduction upon chronic exposure to TiO_2_ nanoparticles. Chemosphere.

[CR17] Weir A, Westerhoff P, Fabricius L, Hristovski K, von Goetz N (2012). Titanium dioxide nanoparticles in food and personal care products. Environmental Science & Technology.

[CR18] Xiong D, Fang T, Yu L, Sima X, Zhu W (2011). Effects of nano-scale TiO_2_, ZnO and their bulk counterparts on zebrafish: acute toxicity, oxidative stress and oxidative damage. Science of the Total Environment.

[CR19] Yang SP, Bar-Ilan O, Peterson RE, Heideman W, Hamers RJ, Pedersen JA (2013). Influence of humic acid on titanium dioxide nanoparticle toxicity to developing zebrafish. Environmental Science & Technology.

[CR20] Zhu, X., Chang, Y., & Chen, Y. (2010a). Toxicity and bioaccumulation of TiO_2_ nanoparticle aggregates in *Daphnia magna*. *Chemosphere, 78*, 209–215. 10.1016/j.chemosphere.2009.11.013 19963236

[CR21] Zhu, X., Wang, J., Zhang, X., Chang, Y., & Chen, Y. (2010b). Trophic transfer of TiO_2_ nanoparticles from daphnia to zebrafish in a simplified freshwater food chain. *Chemosphere, 79*, 928–933. 10.1016/j.chemosphere.2010.03.022 20371096

